# Supplementary material for: Transformation seismology: composite soil lenses for steering surface elastic Rayleigh waves
Source: Sci Rep. 2016 Apr 29;6:25320. doi: 10.1038/srep25320 (PMC4850458; doi:10.1038/srep25320)
Supplement: Supplementary Information [file srep25320-s2.pdf]

SUPPLEMENTARY MATERIAL FOR THE ARTICLE:

Transformation seismology: composite soil lenses for steering surface elastic Rayleigh waves

Andrea Colombi, Sebastien Guenneau, Philippe Roux, and Richard V. Craster

The video attached as supplementary material shows the vertical component of the wavefield propagating along the halfspace for the reference and protected case (color legend is given in Fig. 2). The rooftop displacement as function of time highlights the reduction of the shaking in the protected case.
